# *Cupriavidus metallidurans* Strains with Different Mobilomes and from Distinct Environments Have Comparable Phenomes

**DOI:** 10.3390/genes9100507

**Published:** 2018-10-18

**Authors:** Rob Van Houdt, Ann Provoost, Ado Van Assche, Natalie Leys, Bart Lievens, Kristel Mijnendonckx, Pieter Monsieurs

**Affiliations:** 1 Microbiology Unit, Belgian Nuclear Research Centre (SCK•CEN), B-2400 Mol, Belgium; aprovoos@sckcen.be (A.P.); nleys@sckcen.be (N.L.); kmijnend@sckcen.be (K.M.); pmonsieu@sckcen.be (P.M.); 2 Laboratory for Process Microbial Ecology and Bioinspirational Management, KU Leuven, B-2860 Sint-Katelijne-Waver, Belgium; ado.vanassche@kuleuven.be (A.V.A.); bart.lievens@kuleuven.be (B.L.)

**Keywords:** phenotype microarray, mobile genetic elements, *Cupriavidus*, metal, resistance

## Abstract

*Cupriavidus metallidurans* has been mostly studied because of its resistance to numerous heavy metals and is increasingly being recovered from other environments not typified by metal contamination. They host a large and diverse mobile gene pool, next to their native megaplasmids. Here, we used comparative genomics and global metabolic comparison to assess the impact of the mobilome on growth capabilities, nutrient utilization, and sensitivity to chemicals of type strain CH34 and three isolates (NA1, NA4 and H1130). The latter were isolated from water sources aboard the International Space Station (NA1 and NA4) and from an invasive human infection (H1130). The mobilome was expanded as prophages were predicted in NA4 and H1130, and a genomic island putatively involved in abietane diterpenoids metabolism was identified in H1130. An active CRISPR-Cas system was identified in strain NA4, providing immunity to a plasmid that integrated in CH34 and NA1. No correlation between the mobilome and isolation environment was found. In addition, our comparison indicated that the metal resistance determinants and properties are conserved among these strains and thus maintained in these environments. Furthermore, all strains were highly resistant to a wide variety of chemicals, much broader than metals. Only minor differences were observed in the phenomes (measured by phenotype microarrays), despite the large difference in mobilomes and the variable (shared by two or three strains) and strain-specific genomes.

## 1. Introduction

*Cupriavidus metallidurans* type strain CH34, which was isolated from a decantation basin in the non-ferrous metallurgical factory at Engis, Belgium [1], has been mostly studied because of its resistance to numerous heavy metals [2]. It tolerates high concentrations of metal (oxyan)ions, including Cu^+^, Cu^2+^, Ni^2+^, Zn^2+^, Co^2+^, Cd^2+^, CrO_4_^2−^, Pb^2+^, Ag^+^, Au^+^, Au^3+^, HAsO_4_^2−^, AsO^2−^, Hg^2+^, Cs^+^, Bi^3+^, Tl^+^, SeO_3_^2−^, SeO_4_^2−^ and Sr^2+^ [2,3]. Metal detoxification is encoded by at least 24 gene clusters and many of them are carried by its two megaplasmids pMOL28 and pMOL30 [4]. Resistance to metal ions is mediated by multiple systems, including transporters belonging to the resistance nodulation cell division (RND), the cation diffusion facilitator (CDF) and the P-type ATPase families [2,5].

*Cupriavidus metallidurans* strains have characteristically been isolated from metal-contaminated industrial environments such as soils around metallurgical factories in the Congo (Katanga) and North-Eastern Belgium [6,7], as well as from contaminated soils in Japan [8] and gold mining sites in Queensland (Australia) [9]. Other environments include sewage plants [10], laboratory wastewater (Okayama University, Okayama, Japan) [11] and spacecraft assembly cleanrooms [12]. In addition, *C. metallidurans* strains were also found in the drinking water and dust collected from the International Space Station (ISS) [12,13].

Remarkably, more and more reports describe the isolation of *C. metallidurans* strains from medically-relevant settings and sources such as the pharmaceutical industry, human cerebrospinal fluid and cystic fibrosis patients [14]. It remains to be elucidated if the isolates caused the active infection or only intruded as secondary opportunistic pathogens [14]. Nevertheless, an invasive human infection and four cases of catheter-related infections caused by *C. metallidurans* were recently reported [15,16].

All *Cupriavidus* genomes characteristically carry, next to their chromosome, a second large replicon. This 2 to 3 Mb-sized replicon has recently been coined chromid as it neither fully fits the term chromosome nor plasmid [17,18]. In addition to the chromid, most *Cupriavidus* strains harbor one or more megaplasmids (100 kb or larger in size), which probably mediate the adaptation to certain ecological niches by the particular functions they encode (see [19] for detailed review). For instance, pMOL28 and pMOL30 from *C. metallidurans* CH34 are pivotal in metal ion resistance [4]; hydrogenotrophic and chemolithotrophic metabolism are encoded by pHG1 from *Cupriavidus necator* H16 [20], and pRALTA from *Cupriavidus taiwanensis* LMG19424 codes for nitrogen fixation and legume symbiosis functions [21]. Next to these megaplasmids, other plasmids (mostly broad host range) can be present. One example is pJP4 from *Cupriavidus pinatubonensis* JMP134, which is a broad host range IncP-1β plasmid involved in the degradation of substituted aromatic pollutants [22].

The *C. metallidurans* mobilome is completed with a large diversity of genomic islands (GIs), integrative and conjugative elements, transposons and insertion sequence (IS) elements [7,23,24,25]. Many mobile genetic elements (MGEs) carry accessory genes beneficial for adaptation to particular niches (resistance, virulence, catabolic genes), but acquired genes may also impact the host by cross-talk to host global regulatory networks [26]. In addition, without accessory genes, MGEs such as IS elements can have an impact on genome plasticity and concomitant adaptability of phenotypic traits, including resistance to antibacterial agents, virulence, pathogenicity and catabolism [27]. Finally, the presence of prophages, until now not identified in *C. metallidurans*, may also affect many different traits and lead to phenotypic changes in the host [28,29].

Recently, we showed that *C. metallidurans* strains share most metal resistance determinants irrespective of their isolation type and place [7]. In contrast, significant differences in the size and diversity of their mobilome was observed. However, our comparison was based on whole-genome hybridization to microarrays containing oligonucleotide probes present on the CH34 microarray. These observations triggered us to further study the diversity of the mobilome, its relation to the environment and impact on the host’s global phenome. Therefore, we inventoried the mobilomes and compared the global metabolic capabilities of type strain CH34, strains NA1 and NA4 isolated from water sources aboard ISS [12], and H1130 isolated from an invasive human infection [15]. The global metabolic activities were assessed by employing phenotype microarrays (PMs), which highlight differences in growth requirements, nutrient utilization and sensitivity to chemicals [30].

## 2. Materials and Methods

### 2.1. Strains, Media and Culture Conditions

Bacterial strains and plasmids used in this study are summarized in Table 1. *Cupriavidus metallidurans* strains were routinely cultured at 30 °C in lysogeny broth (LB) or tris-buffered mineral medium (MM284) supplemented with 0.2% (*w/v*) gluconate [1]. *Escherichia coli* strains were routinely cultured at 37 °C in LB. Liquid cultures were grown in the dark on a rotary shaker at 150 rpm. For culturing on agar plates, 1.5% agar (Thermo Scientific, Oxoid, Hampshire, UK) was added. When appropriate, the following chemicals (Sigma-Aldrich (Overijse, Belgium) or Fisher Scientific (Merelbeke, Belgium)) were added to the growth medium at the indicated final concentrations: kanamycin (50 µg/mL for *E. coli* or 1500 µg/mL for *C. metallidurans*), tetracycline (20 µg/mL), 5-bromo-4-chloro-3-indolyl-β-d-galactopyranoside (X-Gal; 40 µg/mL), isopropyl-β-d-thiogalactopyranoside (IPTG; 0.1 mM) and diaminopimelic acid (DAP; 1 mM).

### 2.2. Growth in the Presence of Metals

*Cupriavidus metallidurans* CH34, NA1, NA4 and H1130 were cultivated in MM284 at 30 °C up to stationary phase (10^9^ CFU/mL) and 10 μL of a ten-fold serial dilution in 10 mM MgSO_4_ were spotted on MM284 agar plates containing various metal concentrations (Table S1). Colony forming units (CFU) were counted after 4–5 days. Data are presented as log(N)/log(N_0_) in function of metal concentration, with N and N_0_ CFUs in the presence and absence (control) of metal, respectively.

### 2.3. NA4 CRISPR Deletion Construction

The CRISPR region of *C. metallidurans* NA4 was amplified by PCR (Phusion High-Fidelity DNA polymerase) (Fisher Scientific, Merelbeke, Belgium) with primer pairs CRSPR_Fw-Rv (Table S2), providing XbaI/HindIII restriction sites. Afterwards, this PCR product was cloned as a XbaI/HindIII fragment into the mobilizable suicide vector pK18mob. The resulting pK18mob_CRISPR plasmid from an *E. coli* DG1 transformant selected on LB Km50 was further confirmed by sequencing prior to amplifying of the flanking CRISPR sequences by inverse PCR (Phusion High-Fidelity DNA polymerase) with primer pair CRISPR_tet_Fw-Rv (Table S2), providing BcuI/BspTI restriction sites. At the same time, the *tet* gene from pACYC184 (Table 1 [34]) was amplified by PCR (Phusion High-Fidelity DNA polymerase) with primer pair Tet_Fw-Rv (Supplementary Table S1), providing BcuI/BspTI restriction sites. Afterwards, this PCR product was cloned as a BcuI/BspTI fragment into the former inverse PCR product. The resulting pK18mob-CRISPR::*tet* plasmid from an *E. coli* DG1 transformant selected on LB Tc20 Km50 was further confirmed by sequencing prior to conjugation (with *E. coli* MFDpir as donor host [32]) to *C. metallidurans* NA4. The resulting transformants selected on LB Tc20 were replica plated on LB Tc20 and LB Km1500. NA4 ΔCRISPR::*tet* cells resistant to Tc20 but sensitive to Km1500 were further confirmed by sequencing.

### 2.4. Construction of Plasmids

PCR amplification of *C. metallidurans* CH34 Rmet_2825 was performed on genomic DNA from *C. metallidurans* CH34 with primer pair Rmet2825_Fw-Rv (Table S2). This amplicon was subsequently cloned into pJB3kan1, which was linearized by PCR amplification with the primers pJB3kan1_Fw-Rv (Table S2), using the GeneArt™ Seamless Cloning and Assembly Enzyme Mix (Fisher Scientific, Merelbeke, Belgium). The resulting pJB3kan1-Rmet2825 plasmid from *E. coli* DG1 transformants selected on LB Km50 was further confirmed by sequencing prior to transformation to *E. coli* MFDpir.

### 2.5. Conjugation Assay for Testing CRISPR-Cas

Donor (*E. coli* MFDpir pJB3kan1-Rmet2825) and recipient (*C. metallidurans* NA4 or NA4 ΔCRISPR::*tet*) were grown overnight at 37° in LB Km50 DAP, and at 30° in LB, respectively. Fifty µL of donor and recipient were spotted on a 0.45 µm Supor^®^ membrane disc filter (Pall Life Sciences, Hoegaarden, Belgium) that was put on a LB DAP plate. After overnight incubation at 30 °C, cells were resuspended in 1 mL of 10 mM MgSO_4_ and 10-fold serial diluted on LB Km50 DAP (37 °C), LB (30 °C) and LB Km1500 plates (30 °C) to count CFU of donors, recipients and transconjugants, respectively. Conjugation frequency was measured as the number of transconjugants per donor cell (T/D) and per recipient cell (T/R).

### 2.6. Plasmid Profiling

The extraction of megaplasmids was based on the method proposed by Andrup et al. [36]. Extracted plasmid DNA was separated by horizontal gel electrophoresis (0.5% Certified Megabase agarose gel (Bio-Rad, Temse, Belgium) in 1X TBE buffer, 100 V, 20 h) in a precooled (4 °C) electrophoresis chamber. After GelRed staining (30 min + overnight destaining at 4 °C in ultrapure water), DNA was visualized and images captured under UV light transillumination (Fusion Fx, Vilber Lourmat, Collégien, France).

### 2.7. Phenotype Microarray Analysis

Phenotype microarray (PM) analysis was performed using the OmniLog^®^ automated incubator/reader (Biolog Inc., Hayward, CA, USA) following manufacturer’s instruction (PM procedures for *E. coli* and other GN Bacteria version 16-Jan-06 with slight modifications). Briefly, cells were suspended in Biolog’s inoculation fluid IF-0a (1x) until an optical density (600 nm) of 0.2 was reached. Subsequently, a 1:50 dilution was made in IF-0a (1x) containing dye mix A. Furthermore, 2 mM sodium succinate and 2 µM ferric citrate (Sigma-Aldrich, Overijse, Belgium) were used as carbon sources in PM 3 till 8. All 20 plates (PM-1 through PM-20) inoculated with bacterial cell suspensions, were incubated at 30 °C and cell respiration was measured every 30 min for 144 h. Raw kinetic data were retrieved using the OmniLog—OL_PM_FM/Kin 1.30-: File Management/Kinetic Plot Version software of Biolog. Analysis was carried out with the R-library OPM (version 1.3.64) [37,38]. The area under the curve (AUC) threshold to decide whether a strain is or is not growing in a specific well of the PM, was derived by plotting the AUC values of all PM reactions for each strain, showing in all conditions an almost bimodal distribution. The AUC threshold (one value for all four strains) was determined as the value separating both major peaks (threshold value of 8000) (Figure S1). Negative control wells that contained the inoculated Omnilog™ growth medium without any substrate were measured to normalize differences in inocula and redox dye oxidation between samples.

### 2.8. Computational Methods

The pan-genome analysis was performed via the MaGe platform [39], which uses MicroScope gene families (MICFAM) that are computed with an algorithm implemented in the SiLiX software [40]. The alignment constraints to compute the MICFAM families were 80% amino-acid identity and 80% amino-acid alignment coverage. The MICFAM is part of the core-genome if associated with at least one gene from every compared genome (see Table S3 for complete data set).

A phylogenetic tree of the genomes was constructed via the MaGe platform from the pairwise genome distances using a neighbor-joining algorithm. The pairwise genome distance was calculated with Mash [41].

The CARD (comprehensive antibiotic resistance database) [42] implementation within the MaGe platform [39] was used to identify known resistance determinants and associated antibiotics. All predictions were strict as defined by CARD, meaning a match above the CARD curated bitscore cut-offs [42,43,44].

A BLAST search against BacMet (antibacterial biocide and metal resistance genes database) was used to inventory genes predicted to confer resistance to metals and/or antibacterial biocides [45]. The alignment constraints were 35% amino-acid identity and 80% amino-acid alignment coverage.

The different constraints used to compute the MICFAM families and CARD/BacMet BLAST hits can result in minor differences in the number of core genome genes from a particular strain that results in a positive CARD/BacMet hit.

## 3. Results and Discussion

Four *C. metallidurans* strains were selected: type strain CH34 [3], strain NA1 and NA4 isolated from the drinking water systems onboard the International Space Station that were analyzed previously and had mobilomes divergent from that of CH34 [7], and strain H1130, recently isolated from an invasive human infection [15]. This selection allows comparing the type strain with two strains isolated from a similar environment but with different mobilomes (at least based on elements known in CH34 [7]) and an isolate from a human infection.

### 3.1. Comparison of General Genome Features

**Figure 1:**
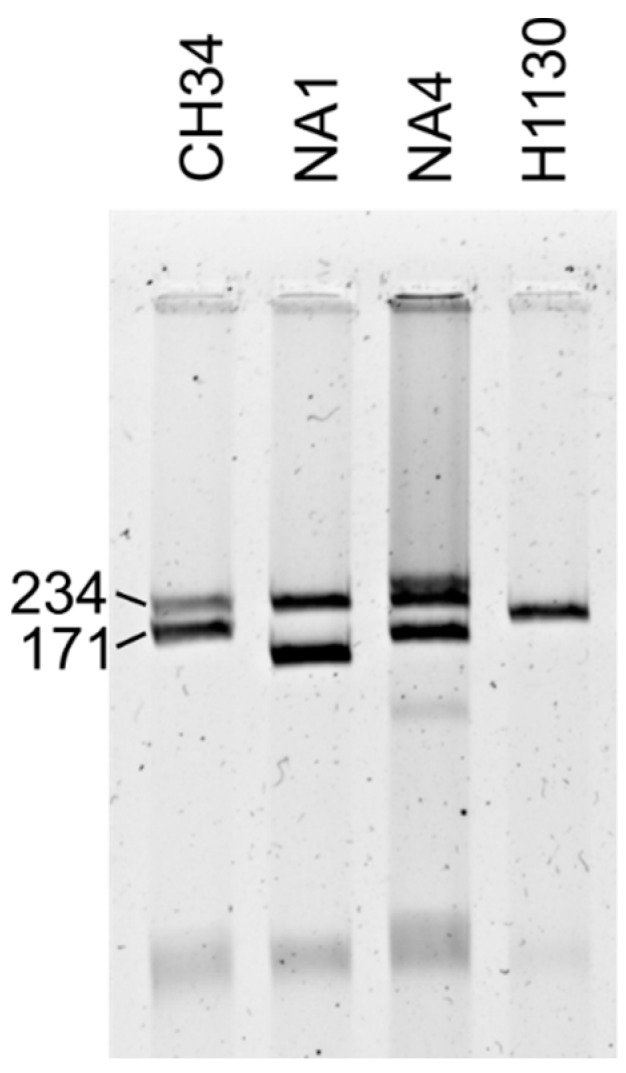
Agarose gel electrophoresis of *Cupriavidus metallidurans* CH34, NA1, NA4 and H1130 (mega)plasmid DNA. The characterized CH34 megaplasmids pMOL30 (234 kb) and pMOL28 (171 kb) serve as reference.

The genome of *C. metallidurans* NA1, NA4 and H1130 was previously sequenced [46,47] and estimated to be 6,833,318 bp, 7,370,364 bp and 7,225,099 bp, respectively (with type strain CH34 being 6,913,352 bp [3]). The G + C content of the genomes are very similar to each other, with 63.76%, 63.27%, 63.50% and 63.82% for NA1, NA4, H1130 and CH34, respectively. NA4 contained the most coding sequences (CDSs) (7467), followed by H1130 (7032), NA1 (6815) and CH34 (6757). All strains contained multiple replicons, namely, one chromosome, one chromid and megaplasmids (>100 kb [19]). Strain NA1 carries two megaplasmids. Strain NA4 carries three megaplasmids and one plasmid. Strain H1130 carries only one megaplasmid (Figure 1).

**Figure 2:**
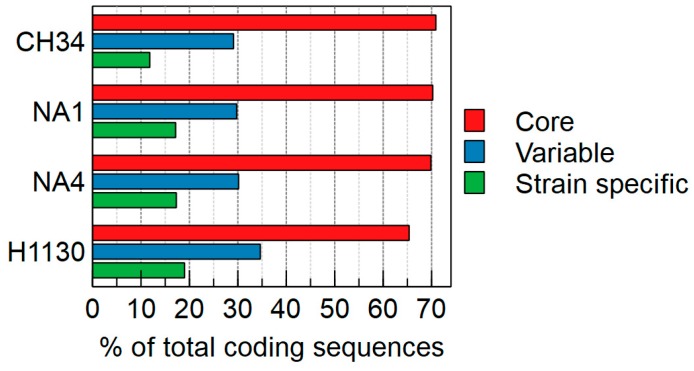
Percentage of coding sequences (CDSs) belonging to the core, variable (shared by two or three strains) and strain-specific genome of *Cupriavidus metallidurans* CH34, NA1, NA4 and H1130.

**Figure 3:**
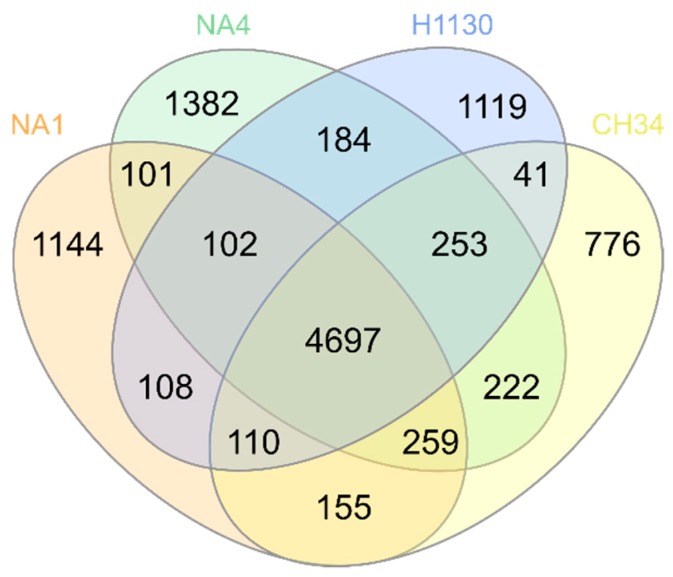
Venn diagram displaying the distribution of shared MicroScope gene families (MICFAM) among *C. metallidurans* CH34, NA1, NA4 and H1130.

**Figure 4:**
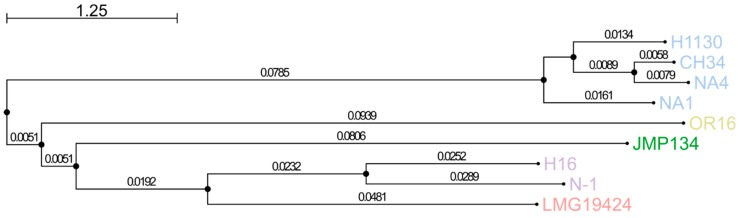
Neighbor-joining phylogenetic tree of *C. metallidurans* CH34, NA1, NA4 and H1130, based on the genome pairwise distance matrix calculated with Mash. *Cupriavidus basilensis*: OR16, *Cupriavidus pinatubonensis*: JMP134, *Cupriavidus necator*: H16 and N-1, and *Cupriavidus taiwanensis*: LMG19424 were included for comparison.

The core genome contains 4697 MICFAM gene families shared by all four strains, which relates to 70.9%, 70.2%, 65.4% and 69.9% of the total CDSs of CH34, NA1, NA4 and H1130, respectively. This means that roughly 30% to 35% of the CDSs belong to the variable (shared by two or three strains) or strain-specific genome (Figure 2). Strains CH34 and NA4 shared the most gene families (Figure 3). Furthermore, the Mash-distance-based phylogeny (Figure 4) indicated that NA4 and CH34 were the most closely related. In addition, NA4 shared more gene families with H1130 and CH34 than with NA1, which corresponded with the phylogenetic distance. These data indicated that NA1 and NA4 were not the two most similar strains, despite their isolation from the same environment.

Evidently, with 1827 Microscope gene families shared with *Cupriavidus taiwanensis* LMG19424, *Cupriavidus necator* H16, *Cupriavidus pinatubonensis* JMP134, *Cupriavidus basilensis* OR16 and *Cupriavidus necator* N-1, the *C. metallidurans* strains share more gene families among each other than with strains of different *Cupriavidus* species. Strains CH34, NA1, NA4 and H1130 shared 1977 gene families unique to the *C. metallidurans* species.

**Figure 5:**
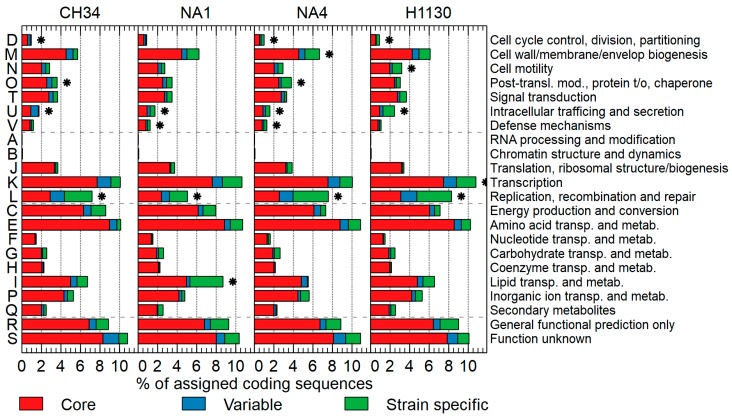
Percentage of CDSs assigned to a COG (clusters of orthologous groups) functional class (general categories: cellular processes and signaling: D, M, N, O, T, U, V; information storage and processing: A, B, J, K, L; metabolism; C, E, F, G, H, I, P, Q; poorly characterized: R, S) belonging to the core, variable of strain-specific genome of *C. metallidurans* CH34, NA1, NA4 and H1130. * Significant (*p* < 0.05; based on hypergeometric distribution) overrepresentation of COG on variable + specific compared to the core genome.

The COGnitor module [48] implemented in the MaGe platform was used to compare the CDSs of the core, variable and specific genome assigned to a COG (clusters of orthologous groups) functional category (Figure 5). The latter indicated that for all four strains, COG L (replication, recombination and repair) and U (intracellular trafficking and secretion) are overrepresented on the variable plus specific genome. Other COGs were also significantly overrepresented on the variable plus specific genome for particular strains. For instance, COG D (cell cycle control, division and partitioning) for CH34, NA4 and H1130, and COG V (defense mechanisms) for NA1 and NA4 (see Figure 5 for all significant overrepresentations).

### 3.2. The Mobilome

Recently, we showed that *C. metallidurans* strains have substantial differences in the diversity and size of their mobile gene pool [7]. However, since this comparison was based on whole-genome hybridization to microarrays containing type strain CH34 oligonucleotide probes, the presence of MGEs other than those in CH34 could not be assessed. Here, the mobilomes of NA1, NA4 and H1130 (including IS elements, transposons, genomic islands and prophages) as well as the presence of CRISPR-Cas systems were scrutinized.

#### 3.2.1. Insertion Sequence Elements and Transposons

ISFinder [49] and ISSaga [50] (+ manual curation) were used to create an inventory of the IS elements, which identified 57, 25, 33 and 91 putative IS elements in CH34 [24], NA1, NA4 and H1130, respectively. It must be noted that this list is based on a draft genome assembly for NA1, NA4 and H1130, which could have an impact on the actual number. Possible identical IS elements present in multiple copies will only be represented as one contig in the genome assembly, as such leading to an underestimation of the number of IS elements in the respective genome [51]. Active IS transposition in CH34 was already observed for IS*Rme1*, IS*Rme3*, IS*Rme5*, IS*Rme15*, IS1086, IS1087B, IS1088 and IS1090 [24,52,53,54,55,56,57,58]. Transposition activity of IS*Rme5* > IS*1088* > IS*Rme3* > IS*1087B* > IS*1090* > IS*1086* > IS*Rme15*, at least into the *cnr* target after exposure of AE126, a derivative of CH34 cured from plasmid pMOL30 carrying the main zinc resistance determinant, to 0.8 mM Zn^2+^ [58]. Some of these active IS elements are also carried by NA1 (2 IS*Rme3* copies), NA4 (1 IS*Rme1*, 4 IS*Rme4* and 1 IS*Rme5* copy) and H1130 (16 IS*Rme3* copies) (based on 98% DNA sequence identity cut-off). Next to transposition, IS elements can also cause more extensive/general loss of genetic information by recombination events between identical individual IS copies, e.g., loss of the CH34 genes involved in autotrophy by IS*1071*-mediated excision [24]. Similar observations of IS*1071*-mediated rearrangements affecting the metabolic potential of the host have been described for *Comamonas* sp. strain JS46 [59] and *Cupriavidus pinatubonensis* JMP134 [60]. Thus, these IS elements in CH34, NA1, NA4 and H1130 can play a multifaceted, pivotal role in the adaptation to stress conditions (as shown for CH34) [27,58].

The CH34 genome harbors five distinct transposon families totaling 19 intact transposons. The transposition modules of four transposons are related to those of mercury transposons with Tn*4378*, Tn*4380* and Tn*6050* belonging to the Tn*21*/Tn*501* family, and Tn*6048* to the Tn*5053* family [61]. The transposition module of Tn*6049* could not be categorized. Tn*6048*, Tn*6049* and mercury transposons are also conserved in NA1 (one Tn*6048* copy, one Tn*6049* copy), NA4 (3 mercury transposons, 3 Tn6049 copies) and H1130 (4 mercury transposons). Tn*6050* appeared to be only present in CH34. No other transposons were identified.

#### 3.2.2. Genomic Islands

The MaGe platform was used to scrutinize the presence of genomic islands (GIs), including those previously identified in CH34. The largest island (109 kb) on the chromosome of CH34 belongs to the large pKLC102/PAGI-2 family of elements that share a core gene set and are integrated downstream of tRNA genes [62,63]. A similar element is present in NA1 (2 copies), NA4 and H1130 as shown by progressive Mauve alignment [64] (Figure S2). The Tn*4371*-family of integrative and conjugative elements CMGI-2, CMGI-3 and CMGI-4 of CH34 were previously designated ICE_Tn*4371*_6054, ICE_Tn*4371*_6055 and ΔICE_Tn*4371*_6056, respectively [65]. CMGI-2 (ICE_Tn*4371*_6054) and CMGI-3 (ICE_Tn*4371*_6055) are responsible for CH34’s ability to grow on aromatic compounds and to fix carbon dioxide, respectively [7,24]. No Tn*4371*-family genomic island was identified in NA4. One Tn*4731*-family element was identified in NA1, which is highly similar to previously identified elements in *Delftia acidovorans* SPH-1 (DAGI-1; ICE_Tn*4371*_60370), *Comamonas testosteroni* KF-1 (CTGI-1; ICE_Tn*4371*_6038) and the partial CMGI-4 (ΔICE_Tn*4371*_6056) of CH34 [25,65]. The island carries an RND-driven efflux system. In H1130, two Tn*4371*-family genomic islands were identified, one carrying 12 genes (putatively involved in ion transport), while the second could not be correctly defined as the integration/excision and stabilization/maintenance module up to *rlxS* (encoding a relaxase protein) are not located on the same contig as the transfer module (starting from *traR* coding for a transcriptional regulator). Therefore, the accessory genes that are typically located between *rlxS* and *traR* in Tn*4371*-family members could not be properly assessed [65]. All other GIs on CH34’s chromosome were not found in the other strains, except CMGI-5 in NA1. CMGI-C and CMGI-E, previously identified on CH34’s chromid, are absent in all strains. CMGI-A, -B and -D are conserved in NA4 and H1130, but show limited synteny with NA1. No other genomic islands could be clearly identified in NA1 or NA4. One other genomic island was clearly noticeable in H1130. This 87 kb region, which is absent in CH34, NA1 and NA4, is syntenic with an 80-kb cluster located on the 1.47-Mbp megaplasmid of *Burkholderia xenovorans* LB400. In *B. xenovorans* LB400, this Dit island encodes proteins of abietane diterpenoids metabolism and mediates growth on abietic acid, dehydroabietic acid, palustric acid and 7-oxo-dehydroabietic acid [66] (not included in the phenotypic microarray). Abietane diterpenoids are tricyclic, C-20, carboxylic acid-containing compounds produced by plants and are a key component of the defense systems of coniferous trees [66,67]. This observation also adds evidence to the mobility of this cluster and its distribution among proteobacterial genomes [66]. In addition, two smaller regions (13.6 and 10.3 kb) carrying genes coding for unknown functions and a tyrosine-based site-specific recombinase were identified.

#### 3.2.3. Prophages

The presence of prophages was scrutinized via PHASTER [68] and showed no prophages in type strain CH34 (which was already known) and the presence of intact prophages in NA4 and H1130 as well as incomplete/remnants in H1130, NA1 and NA4 (Table 2). Although mitomycin C exposure did not result in prophage induction (data not shown), a derivative of NA4 exposed to uranium lost the 43.6 kb region predicted as an intact prophage (unpublished data).

#### 3.2.4. CRISPR-Cas

**Figure 6:**
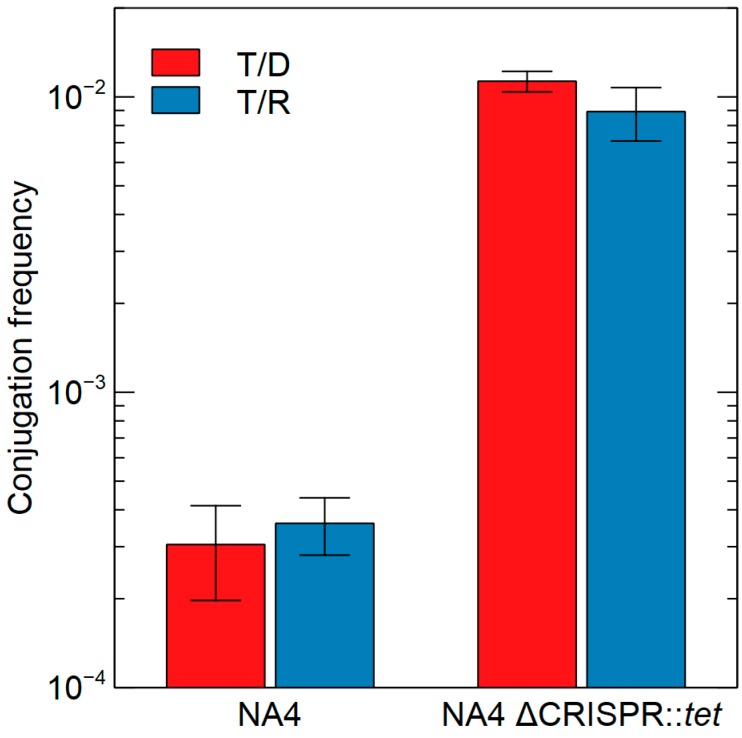
Conjugation frequency of pJB3kan1_Rmet2825 (containing one spacer identified in the NA4 CRISP-Cas system) from donor *E. coli* MFDpir to recipient *C. metallidurans* NA4 and NA4 ΔCRISPR::*tet*, respectively. Median values plus corresponding calculated standard deviations across biological triplicates are shown (T/D = conjugation frequency per donor; T/R = conjugation frequency per recipient).

The CRISPR-Cas system is an adaptive immunity system that stores memory of past encounters with foreign DNA in spacers that are inserted between direct repeats in CRISPR arrays [69]. CRISPR-Cas systems were detected with CRISPRfinder [70] and CRISPRDetect [71] (default settings). Only positive hits with both were further examined, resulting in the identification of 1 CRISPR-Cas system in NA4. CRISPRTarget [72] identified 5 spacer sequences related to genomic island CMGI-5 of CH34 (which is also present in NA1). CMGI-5 is probably a plasmid remnant and contains besides hypothetical genes, some typical plasmid-related genes such as *repA*, *traY*, *mobA* and *mobB*. To assess if the identified system is active, the conjugation frequency of plasmid pJB3kan1 carrying the CMGI-5 *repA* gene (pJB3kan1_Rmet2825; containing one spacer) was determined for the parental and CRISPR-deleted NA4 strain. CRISPR deletion in NA4 increased the conjugation efficiency 33-fold, indicating an active CRISPR-Cas system in NA4 (Figure 6).

### 3.3. The Resistome

#### 3.3.1. Antibiotic Resistance

The CARD [42] implementation within the MaGe platform [39] was used to identify known resistance determinants and associated antibiotics. The latter predicted 33, 36, 33 and 39 proteins involved in antibiotic resistance in CH34, NA1, NA4 and H1130, of which 31, 31, 30 and 39 belonged to the core genome, respectively. No marked difference in tolerance to antibiotics was observed.

#### 3.3.2. Metal Resistance

**Figure 7:**
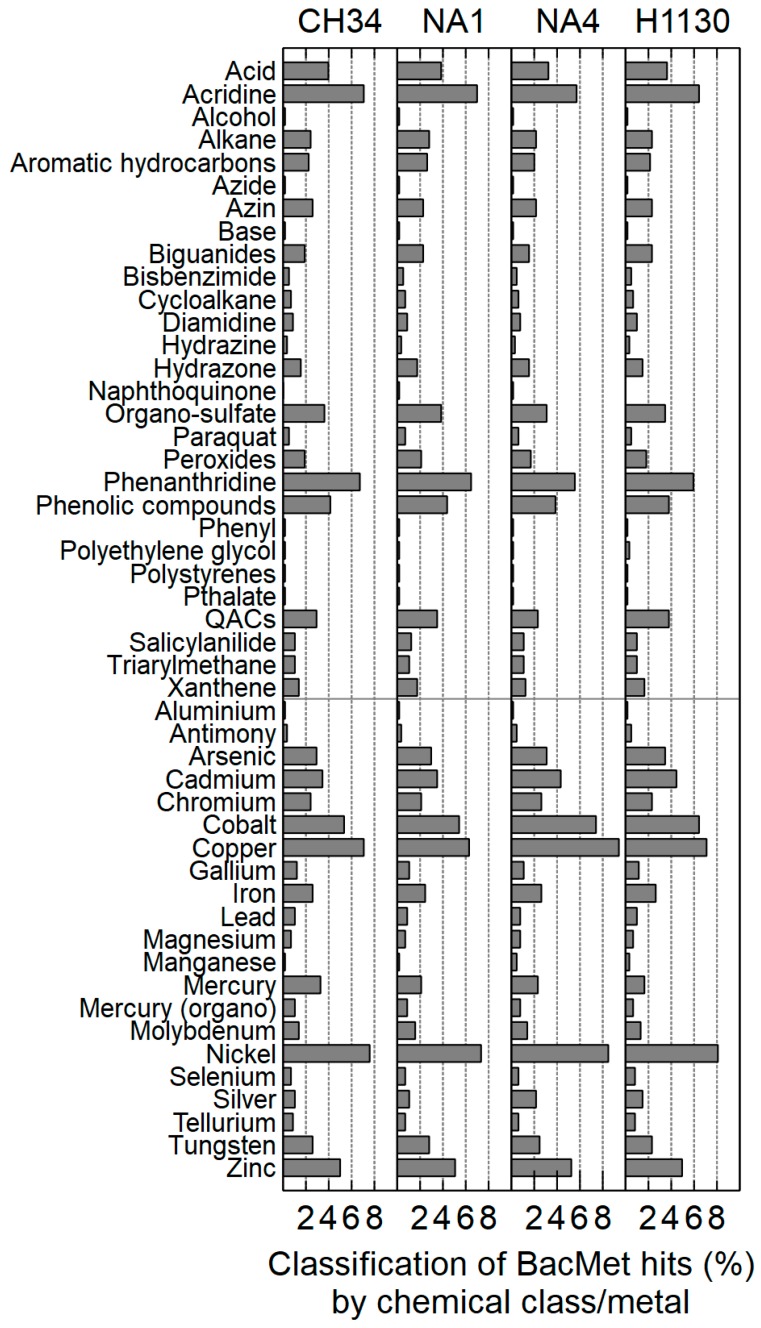
Inventory of *C. metallidurans* CH34, NA1, NA4 and H1130 genes conferring resistance to metals/chemical classes based on the BacMet database (antibacterial biocide and metal resistance genes database).

The antibacterial biocide and metal resistance genes database (BacMet) was used to create an inventory of genes predicted to confer resistance to metals and/or antibacterial biocides [45]. This showed 302, 282, 337 and 302 proteins involved in biocide and metal resistance in CH34, NA1, NA4 and H1130, respectively. Most genes belonged to the core genome (221, 246, 276 and 251 for CH34, NA1, NA4 and H1130, respectively). The compounds (metal and chemical class) to which these genes confer resistance are very similar for all four strains (Figure 7). Genes conferring resistance to nickel, copper, cobalt and the chemical classes acridine and phenanthridine were the most abundant.

For CH34, the predicted genes contained 68 out of the 174 genes that were previously identified to be related to metal resistance (for an overview see [2,3]). Specific analysis of these 174 proteins showed that almost all are conserved in NA1, NA4 and H1130. Exceptions are (*i*) the accessory cluster related to chromate resistance in H1130, (*ii*) the *hmz* cluster in NA4 and H1130, (*iii*) *cdfX* in NA1, NA4 and H1130, and (*iv*) the *dax*/*gig* cluster in NA1. The latter three are all located on a genomic island. The gene cluster related to chromate resistance on pMOL28 from CH34 contains five additional genes that are strongly induced by chromate in CH34 [73] as well as for the homologous system in *Arthrobacter* sp. FB24 (both at the gene and protein level) [74,75]. The *hmz* cluster is a HME-RND-driven system, belonging to the HME3b (Heavy Metal Efflux) subfamily of the RND superfamily, with no known substrate and transcriptionally silent in *C. metallidurans* CH34 [5,73,76]. The *cdfX* gene of CH34 encodes a putative permease (211 amino acid residues and six predicted transmembrane α-helices) that shares 87% amino-acid identity with PbtF from *Achromobacter xylosoxidans* A8 [5]. Expression of *pbtF* in *A. xylosoxidans* A8 was induced by Pb^2+^, Cd^2+^ and Zn^2+^, and although PbtF showed measurable Pb^2+^-efflux activity, it did not confer increased metal tolerance in *E. coli* GG48 [77]. The *dax* cluster [73], which was renamed *gig* for “gold-induced genes” in Wiesemann et al. [78], is induced by Ag^+^ and Au^3+^ [73,79] but not essential for gold resistance [78].

**Figure 8:**
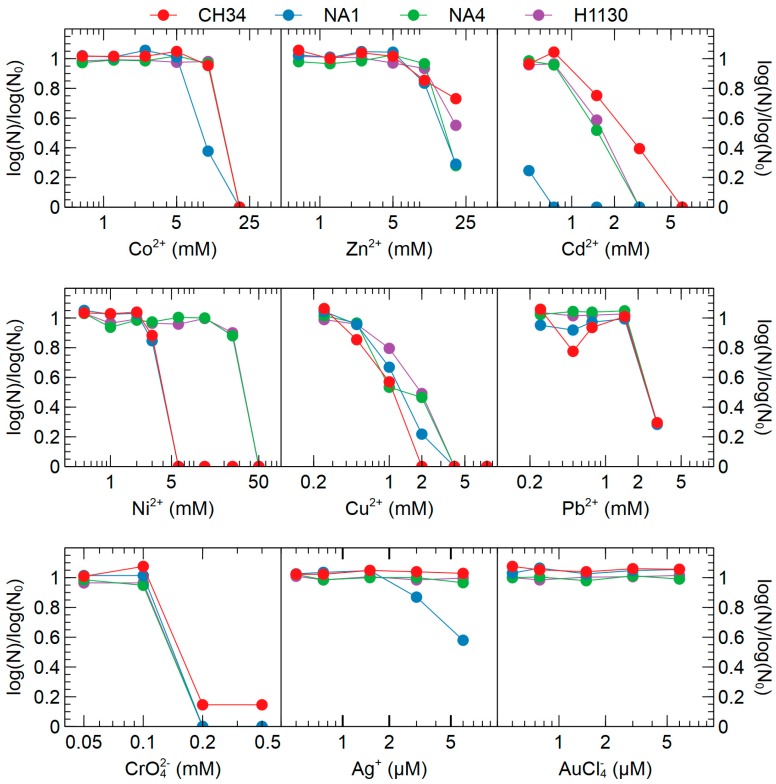
Viable count of *C. metallidurans* CH34, NA1, NA4 and H1130 grown in the presence of various metal concentrations (Table S2). Data are presented as log(N)/log(N_0_) in function of metal concentration, with N and N_0_ the colony forming units (CFUs) in the presence and absence (control) of metal, respectively.

In agreement with the conservation of these metal resistance determinants, growth in the presence of increasing metal concentrations showed only minor differences between CH34, NA1, NA4 and H1130 (Figure 8). Moreover, the minor strain-dependent differences (see above) did not mediate differences in metal resistance (Figure 8). Essentially, the most noticeable difference in growth was observed in the presence of Ni^2+^, with higher concentrations tolerated by NA4 and H1130. Initially, the *nccCBA* locus, which is inactivated in CH34 because of a frame shift mutation, was put forward as a possible explanation [12]. However, the frame shift mutation in *nccB* is present in all four strains. However, NA4 and H1130 carry a second *nccYXHCBAN* locus coding for an RND-driven efflux system involved in Ni^2+^ and Co^2+^ resistance. This locus is homologous to that of *C. metallidurans* 31A and KT02, which has been shown to be responsible for resistance to 40 mM Ni^2+^ [80], and is likely responsible for the observed differences. In addition, although the *nimBAC* locus, coding for an RND-driven efflux system putatively involved in Ni^2+^ and Co^2+^ resistance [5], is only inactivated in CH34 (via IS*Rme3* insertion) and not in NA1, NA4 and H1130, growth in the presence of Ni^2+^ is similar for NA1 and CH34. Other observations are the lower resistance of NA1 to Cd^2+^ and to lesser extent Co^2+^ and Ag^+^. However, based on the current data, no hypotheses can be put forward to explain these observations.

### 3.4. Phenotypic Microarrays

**Figure 9:**
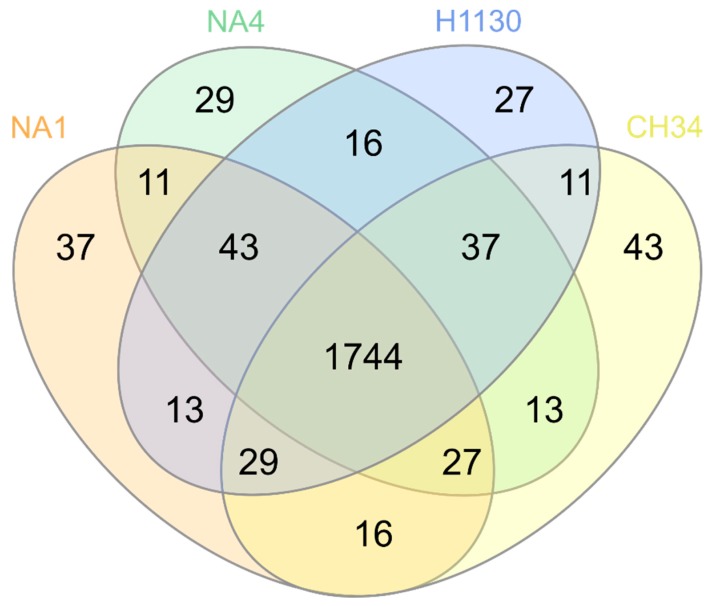
Venn diagram displaying the distribution of OmniLog phenotypic assays shared among *C. metallidurans* CH34, NA1, NA4 and H1130.

**Figure 10:**
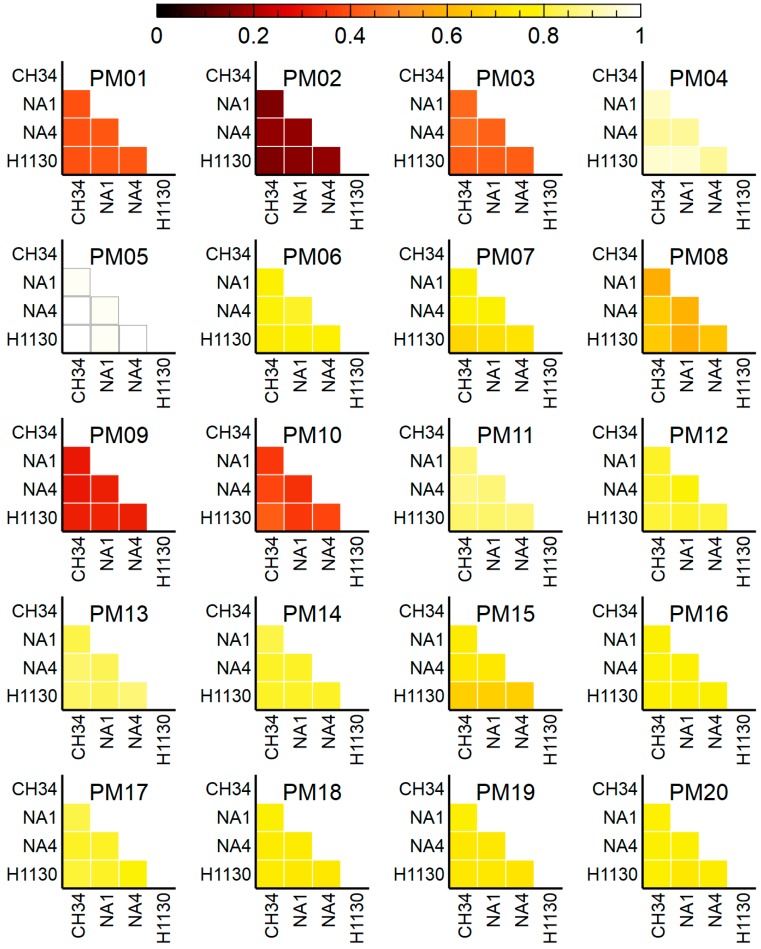
Overview of positive (growth) OmniLog phenotypic assays shared by *C. metallidurans* CH34, NA1, NA4 and H1130 for each PM plate (with 1 being all 96 assays). The assays on each PM plate are detailed in Table S4.

**Figure 11:**
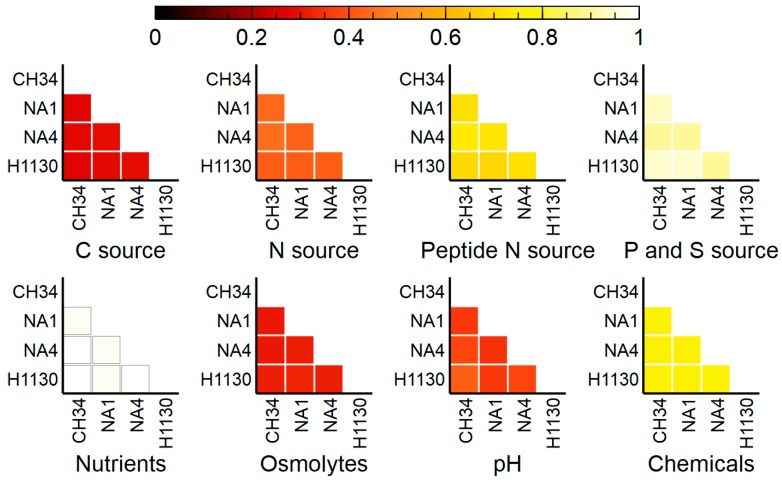
Overview of positive (growth) OmniLog phenotypic assays shared by *C. metallidurans* CH34, NA1, NA4 and H1130 for different metabolic and chemical sensitivity tests (with 1 being all assays shared).

In order to scrutinize functional differences between the four *C. metallidurans* strains, phenotypic characterization with OmniLog Phenotypic Microarrays (PMs) was conducted. Area under the curve (AUC) values were calculated and a threshold cut-off (8000) was applied to discriminate a positive (growth) from a negative (no-growth) reaction. This revealed an overall phenotypic similarity among the four strains, with 1744 out of the 1920 assays shared (Figure 9, Figure 10 and Figure 11).

#### 3.4.1. C, N, P and S Sources

Only around 27% to 28% of the C source reactions was positive, which is related to their inability to assimilate sugars and sugar alcohols (Figure 11) [1,3]. All four strains lack a glucose uptake system. The latter is most likely deleted in all four strains as a N-acetylglucosamine-specific phosphotransferase system (PTS)-type transport system essential for glucose uptake (growth) in *Cupriavidus necator* H16 [81,82] is absent in from a large syntenic region (>110 genes) conserved among *C. necator* H16 and *C. metallidurans* CH34, NA1, NA4 and H1130 (data not shown).

**Figure 12:**
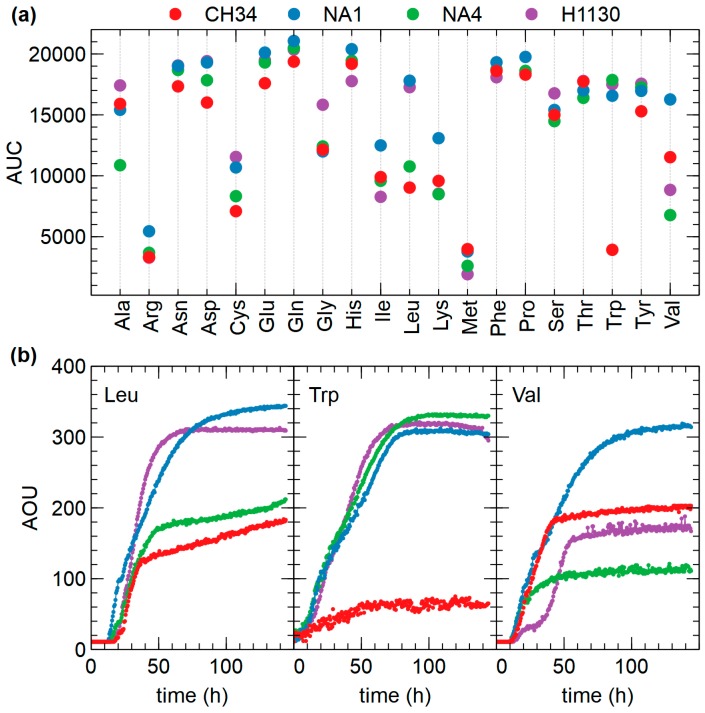
(**a**) Overview of OmniLog phenotypic assays with amino acids as N source (AUC = area under the curve) for *C. metallidurans* CH34, NA1, NA4 and H1130, (**b**) Growth kinetics in the presence of L-leucine, L-tryptophan and L-Valine as N source (AOU = arbitrary OmniLog units).

A few marked differences were observed for the use of amino acids as N source, in particular for L-leucine, L-tryptophan and L-Valine (Figure 12). Specific for L-tryptophan, growth was observed for NA1, NA4 and H1130 but not for CH34. Aerobic L-tryptophan degradation in *C. metallidurans* most likely occurs via a three-step pathway to anthrilanate requiring tryptophan 2,3-dioxygenase (*kynA*), kynurenine formamidase (*kynB*) and kynureninase (*kynU*). Experimental verification of the anthranilate pathway was achieved by functional expression of the CH34 *kynBAU* operon in *Escherichia coli* after suppressing the stop codon disrupting *kynB* [83]. This amber mutation is not present in NA1, NA4 and H1130, which could explain the observed differences. Similar differences were also observed when growth was scored for dipeptides (N source), as CH34 grew less or not on L-tryptophan-containing dipeptides compared to NA1, NA4 and H1130. Only minor differences were observed for growth on P and S sources (Figure 11, Table S4).

#### 3.4.2. Osmolytes and pH

The addition of ionic osmolytes had a clear and comparable impact on the growth of strains CH34, NA1, NA4 and H1130, as growth was generally only observed for the lower/lowest concentrations (1% NaCl, 2% Na_2_SO_4_, 1% sodium formate, 3% urea and 2% sodium lactate). In contrast, addition of up to 20% of the non-ionic osmolyte ethylene glycol had no impact on growth of CH34, NA1, NA4 and H1130.

**Figure 13:**
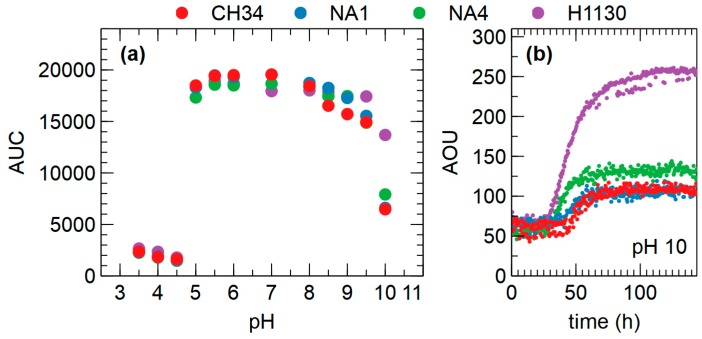
(**a**) Overview of OmniLog phenotypic assays related to pH (AUC = area under the curve) for *C. metallidurans* CH34, NA1, NA4 and H1130, (**b**) Growth kinetics at pH 10 (AOU = arbitrary OmniLog units).

The effect of pH over the range 3.5 to 10 growth was comparable for CH34, NA1, NA4 and H1130. Growth was inhibited below pH 5 for all strains. Growth at pH 10 was much more pronounced for H1130 than for the other strains (Figure 13).

#### 3.4.3. Chemicals

**Figure 14:**
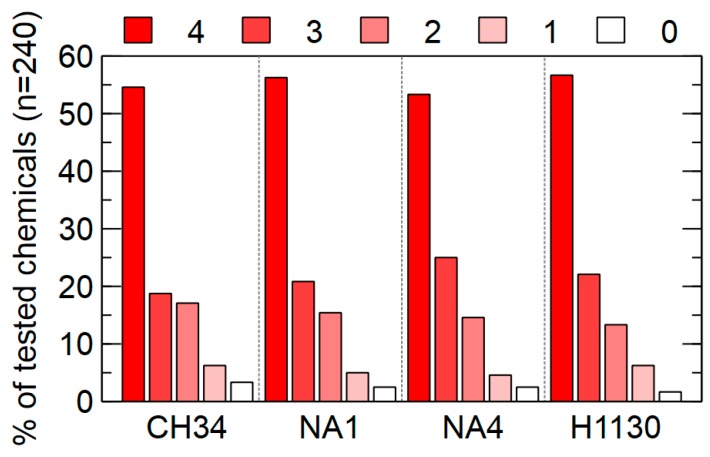
Percentage of tested chemicals (n = 240) to which *C. metallidurans* CH34, NA1, NA4 and H1130 are resistant. Four increasing concentrations are included in the phenotypic microarrays (0: susceptible to the lowest concentration, 1 to 4: resistant to the lowest up to the highest concentration).

The PM-11 to PM-20 plates carry different chemicals (4 increasing concentrations of each) to test sensitivity, only for eight out of the 240 chemicals tested at least one of the strains was susceptible to the lowest concentration. For more than 50% of the tested chemicals, CH34, NA1, NA4 and H1130 were resistant to the highest concentration included in the phenotypic microarrays (Figure 14).

**Figure 15:**
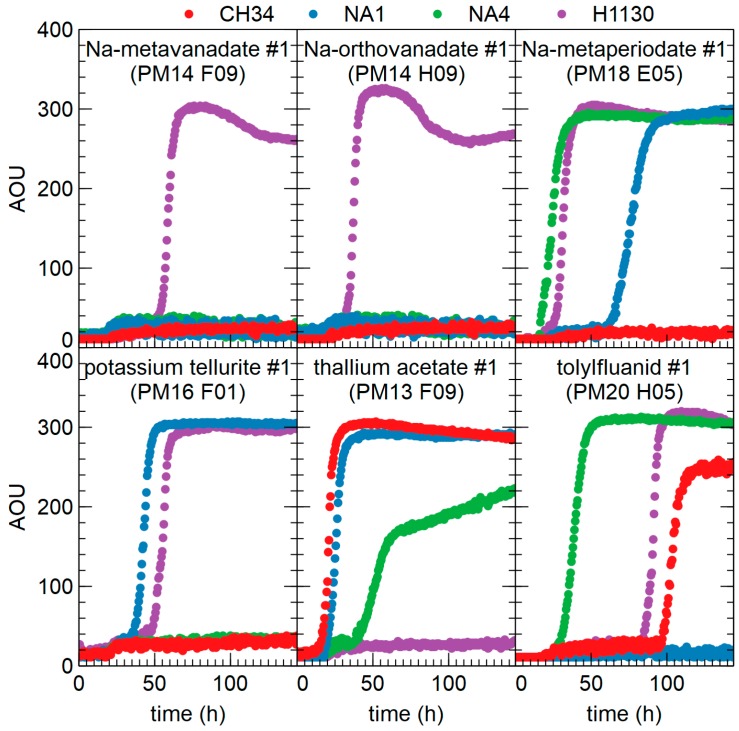
Growth kinetic of *C. metallidurans* CH34, NA1, NA4 and H1130 in the presence of different chemicals (lowest concentration in the phenotypic microarrays is shown) (AOU = arbitrary OmniLog units).

No growth was observed in the presence of 2,2′-dipyridyl (metal chelator), hydroxyurea (ROS producer) and phenethicillin (a narrow-spectrum, β-lactamase-sensitive penicillin) for all four strains. In contrast to phenethicillin, CH34, NA1, NA4 and H1130 were resistant to (at least one concentration of) all other β-lactam antibiotics tested. Only H1130 grew in the presence of sodium meta- and orthovanadate, and did not grow in the presence of thallium acetate (Figure 15). Strain CH34 and NA4 did not grow in the presence of potassium tellurite (Figure 15). The genetic basis underlying resistance to these metals is poorly understood, therefore, no correlation to the genotype could be established. Strain CH34 and NA1 were susceptible to sodium metaperiodate (oxidizing agent) and tolylfluanid (fungicide), respectively (Figure 15).

#### 3.4.4. Trait Prediction

**Figure 16:**
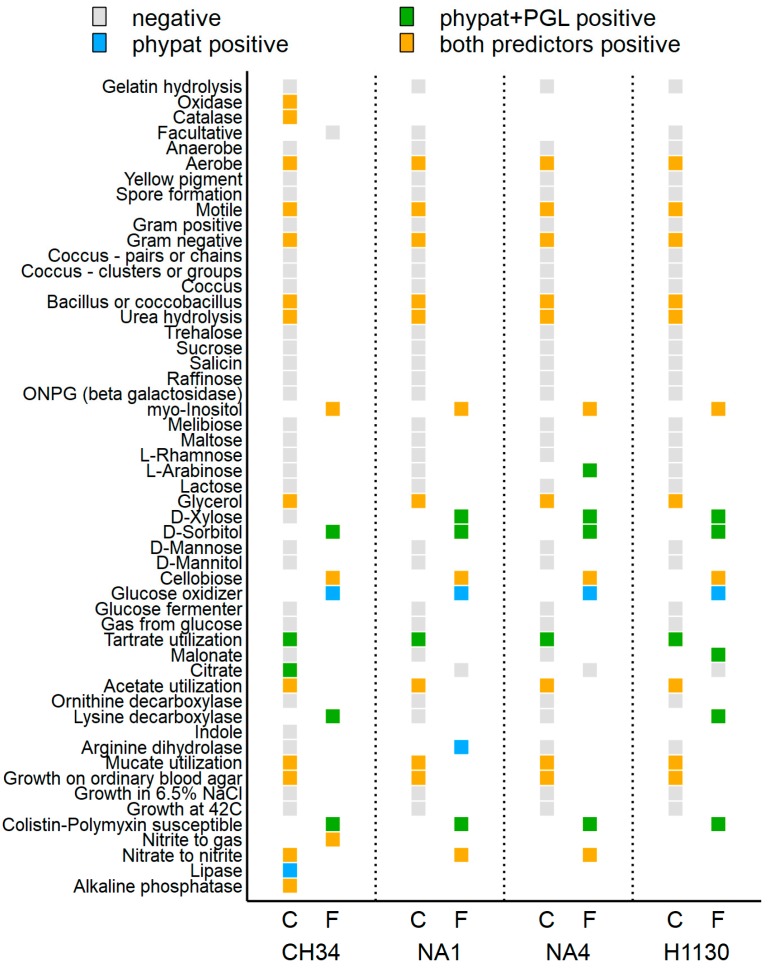
Overview of the correctly (C) and falsely (F) predicted phenotypic traits of *C. metallidurans* CH34, NA1, NA4 and H1130 by Traitar (two classifiers: phypat and phypat + PGL).

Finally, the prediction of Traitar, an automated software framework for the accurate prediction of 67 phenotypes directly from a genome sequence [84], was evaluated by comparison with the generated phenotypic data (OmniLog Phenotypic Microarray data and previous observations/knowledge). Traitar correctly predicted 85% (45 out of 53 analyzed), 81% (38 out of 47), 80% (37 out of 46) and 80% (37 out of 46) of the CH34, NA1, NA4 and H1130 traits, respectively (Figure 16). Although Weimann and colleagues [84] indicated that the phypat classifier assigned more phenotypes at the price of more false-positive predictions, whereas the phypat + PGL classifier assigned fewer phenotypes with fewer false assignments, it appeared that in the case of the *C. metallidurans* strains, phypat + PGL assigned more false-positive predictions.

## 4. Conclusions

The comparison of four *C. metallidurans* strains isolated from different environments indicated that metal resistance determinants and properties are maintained in these environments. As most of the metal determinants are on the native megaplasmids, it could be argued that these environments provided a selective pressure for the conservation of these determinants and plasmids. The previously identified differences in the size and diversity of the mobile gene pool were put in perspective by the identification of intact (and remnant) prophages in NA4 and H1130, and a genomic island putatively involved in abietane diterpenoids metabolism in H1130. The latter indicated that mobilome diversity differed (integrative and conjugative elements/genomic islands versus prophages). Furthermore, the mobilome is apparently not directly related to the isolation environment as the NA1 mobilome is shaped more like that of H1130 than that of NA4 isolated from the same environment. In addition, an active CRISPR-Cas system was identified in strain NA4, providing immunity to a plasmid that integrated in CH34 and NA1. Despite the large size of the variable and specific genomes, only minor differences were observed in the global phenomes (as measured by phenotype microarrays) and all four strains were highly resistant to a wide variety of chemicals, much broader than metals. The variable and specific genome were probably acquired through later transfer and perhaps carry functions more essential for survival in challenging and fluctuating environments than general metabolic functions.

## Figures and Tables

**Table 1:** Strains and plasmids used in this study.

Strain or Plasmid	Genotype/Relevant Characteristics	Reference
STRAIN		
*Cupriavidus metallidurans*		
CH34^T^	Type strain	[31]
NA1	Isolated from a water sample, ISS	[12]
NA4	Isolated from a water sample, ISS	[12]
NA4 ΔCRISPR	ΔCRISPR::*tet*, Tc^R^	This study
H1130	Isolated from invasive human infection	[15]
*Escherichia coli*		
DG1	*mcrA* Δ*mrr-hsdRMS-mcrBC* (r_B_^−^m_B_^−^) Φ80*lacZ*Δ*M15* Δ*lacX74 recA1 araD139* Δ(*ara-leu*)*7697 galU galK rpsL endA1 nupG*	Eurogentec
MFDpir	MG1655 RP4-2-Tc::[Δ*Mu1*::*aac(3)IV*-Δ*aphA*-Δ*nic35*-Δ*Mu2*::*zeo*] Δ*dapA*::(*erm-pir*) Δ*recA*	[32]
PLASMID		
pK18mob	pMB1 ori, *mob*+, *lacZ*, Km^R^	[33]
pK18mob-CRISPR	CRISPR region of NA4 in pK18mob, Km^R^	This study
pK18mob-CRISPR::*tet*	pK18mob-CRISPR derivative, CRISPR::*tet*, Km^R^, Tc^R^	This study
pACYC184	p15A ori, Cm^R^, Tc^R^	[34]
pJB3kan1	RK2 minimal replicon; Ap^R^, Km^R^	[35]
pJB3kan1_Rmet2825	Rmet_2825 of CH34 in pJB3kan1; Km^R^	This study

Eurogentec: Seraing, Belgium, Km^R^: kanamycine resistant, Tc^R^: tetracycline resistant, Cm^R^: chloramphenicol resistant, Ap^R^: ampicillin resistant.

**Table 2:** Prophage detected in *C. metallidurans* NA1, NA4 and H1130.

Strain	Size ^a^	Completeness ^b^	Score ^c^	# ^d^	Position	Most Common Phage ^e^	GC %
NA1	27.9	questionable	90	32	528,474–556,451	Ralsto_RS138 (NC_029107; 7)	65.35
	17.7	incomplete	20	21	554,542–572,263	Pseudo_NP1 (NC_031058; 3)	64.23
NA4	43.6	intact	100	50	1,706,628–1,750,233	Bordet_BPP_1 (NC_005357; 18)	64.94
	6.1	intact	100	10	1,941,664–1,947,835	Ralsto_PE226 (NC_015297; 6)	60.08
	45.2	intact	150	41	2,145,854–2,191,126	Burkho_Bcep176 (NC_007497; 11)	61.83
	8	incomplete	30	10	2,181,367–2,189,450	Gordon_Nymphadora (NC_031061; 2)	62.44
	120.5	intact	130	125	2,248,504–2,369,042	Salmon_118970_sal3 (NC_031940; 14)	61.84
	44.5	incomplete	30	39	2,545,435–2,589,959	Pseudo_JBD44 (NC_030929; 5)	63.89
H1130	19.3	incomplete	30	21	1,470,543–1,489,908	Burkho_phiE125 (NC_003309; 3)	61.27
	12.7	incomplete	40	19	1,505,965–1,518,710	Bacill_SP_15 (NC_031245; 5)	63.39
	7.9	incomplete	30	9	2,763,200–2,771,108	Entero_phi92 (NC_023693; 4)	58.60
	48.4	intact	110	73	6,748,679–6,797,173	Salmon_SEN34 (NC_028699; 22)	62.09
	16	incomplete	50	29	7,110,429–7,126,525	Clostr_phiCT453B (NC_029004; 4)	61.04

^a^ Size in kb; ^b^ Prediction of whether the region contains an intact or incomplete prophage and ^c^ score based on PHASTER criteria [68]; ^d^ number of proteins; ^e^ the phage with the highest number of proteins most similar to those in the region (between parentheses: accession number; number of proteins).

## Supplementary Materials

The following are available online at http://www.mdpi.com/2073-4425/9/10/507/s1, Figure S1: Density plot of the AUC values of all PM reactions for each strain, Figure S2: Progressive Mauve alignment of CMGI-1 of CH34 with related elements in NA1, NA4 and H1130, Table S1: Metal concentrations used in growth experiments, Table S2: Primers used in this study, Table S3: Pan-genome analysis data set, Table S4: PM area under the curve (AUC) values.
