# Supplementary material for: Cupriavidus metallidurans Strains with Different Mobilomes and from Distinct Environments Have Comparable Phenomes
Source: Genes (Basel). 2018 Oct 18;9(10):507. doi: 10.3390/genes9100507 (PMC6210171; doi:10.3390/genes9100507)
Supplement: Supplementary file 1 [file genes-09-00507-s001.zip › S1_Table.docx]

**Table S1.** Metal concentrations used in growth experiments.

| Cd^2+^  (mM) | Zn^2+^  (mM) | Ni^2+^  (mM) | Co^2+^  (mM) | Cu^2+^  (mM) | Pb^2+^  (mM)^1^ | CrO_4_^2-^  (mM) | Ag^+^  (µM) | Au^3+^  (µM) |
| --- | --- | --- | --- | --- | --- | --- | --- | --- |
| 6 | 20 | 50 | 20 | 8 | 3 | 0.4 | 6 | 6 |
| 3 | 10 | 25 | 10 | 4 | 1.5 | 0.2 | 3 | 3 |
| 1.5 | 5 | 12 | 5 | 2 | 0.75 | 0.1 | 1.5 | 1.5 |
| 0.75 | 2.5 | 6 | 2.5 | 1 | 0.5 | 0.05 | 0.75 | 0.75 |
| 0.5 | 1.25 | 3 | 1.25 | 0.5 | 0.25 |  | 0.5 | 0.5 |
|  | 0.625 | 2 | 0.625 | 0.25 | 0.125 |  |  |  |
|  |  | 1 |  |  |  |  |  |  |
|  |  | 0.5 |  |  |  |  |  |  |

^1^For testing Pb^2+^ resistance, cells were grown on RM medium, a modified MM284 in which Tris-HCl is replaced by 20 mM morpholinepropanesulfonic acid (MOPS)–NaOH (pH 7) and PO_4_^2-^ is replaced by 0.95 mM beta-glycerol-phosphate.
