# Supplementary material for: Cupriavidus metallidurans Strains with Different Mobilomes and from Distinct Environments Have Comparable Phenomes
Source: Genes (Basel). 2018 Oct 18;9(10):507. doi: 10.3390/genes9100507 (PMC6210171; doi:10.3390/genes9100507)
Supplement: Supplementary file 1 [file genes-09-00507-s001.zip › S2_Table.docx]

**Table S2.** Primers used in this study.

| **Primer** | **5’-3’ sequence^1^** |
| --- | --- |
| CRSPR_Fw | GATCTCTAGACATGCCGAGTTTCCAGTACA |
| CRSPR_Rv | GATGAAGCTTGTAGGAACCGCCGATCAATA |
| CRSPR_tet_Fw | GATCCTTAAGAGCGATAGCCATACCGGCCA |
| CRSPR_tet_Rv | GATCACTAGTAAGCGTGCAGCATATCGCGC |
| Tet_Fw | GATCACTAGTTCAGCCCCATACGATATAAG |
| Tet_Rv | TTATCTTAAGTGGAGTGGTGAATCCGTTAG |
| pJB3kan1_Fw | GCTTGATCTGTGGAATTGTGAGCG |
| pJB3kan1_Rv | ATTCGACTAGATCTGCTGGCGAA |
| Rmet2825_Fw | AGTCGAATTCCGACGACAGTAGGGGAAGAA |
| Rmet2825_Rv | GATCAAGCTTCGCATCCAGAGTCTATGCAA |

^1^Restriction sites are underlined.
