# Supplementary figures and images for: Cupriavidus metallidurans Strains with Different Mobilomes and from Distinct Environments Have Comparable Phenomes
Source: Genes (Basel). 2018 Oct 18;9(10):507. doi: 10.3390/genes9100507 (PMC6210171; doi:10.3390/genes9100507)

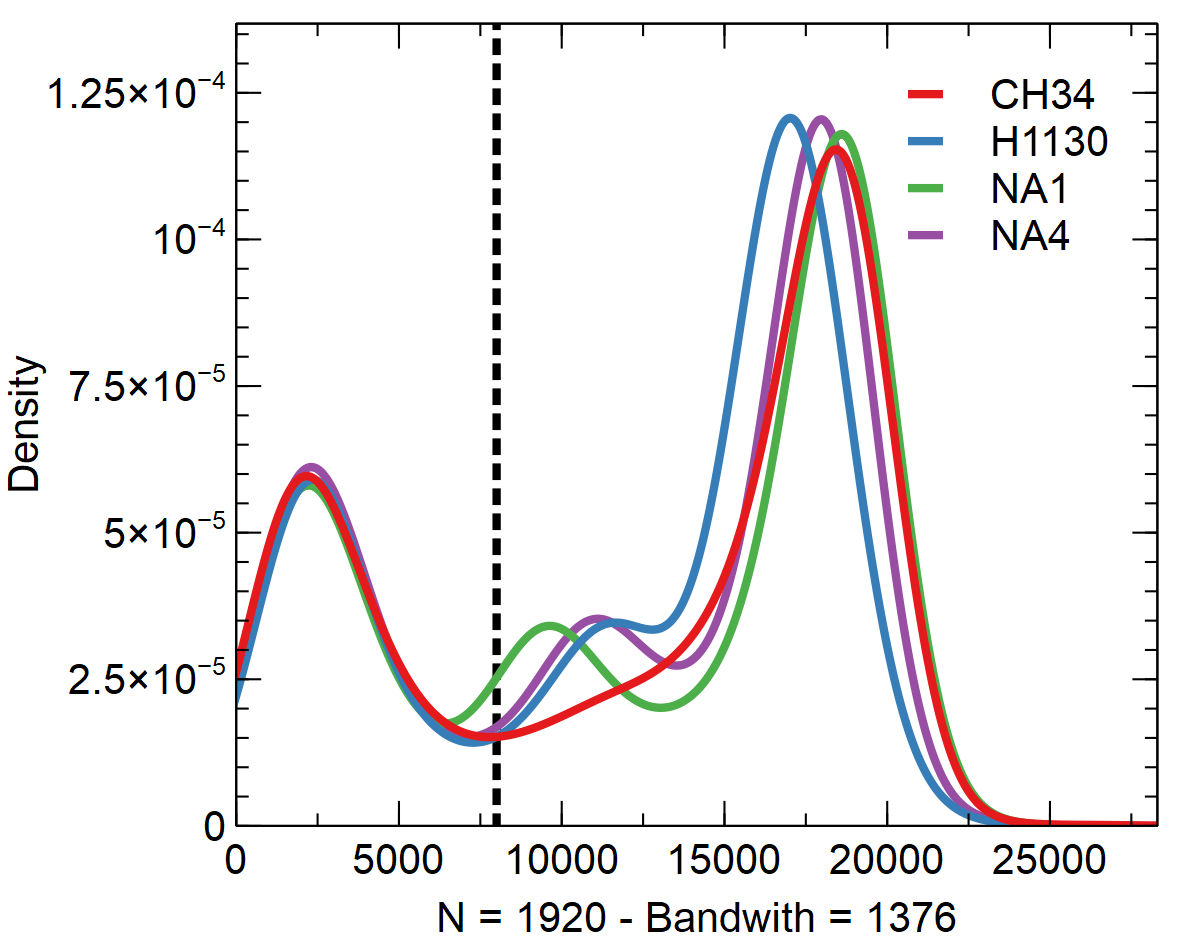

Supplement: Supplementary file 1 [file genes-09-00507-s001.zip › S1_Figure.tiff]

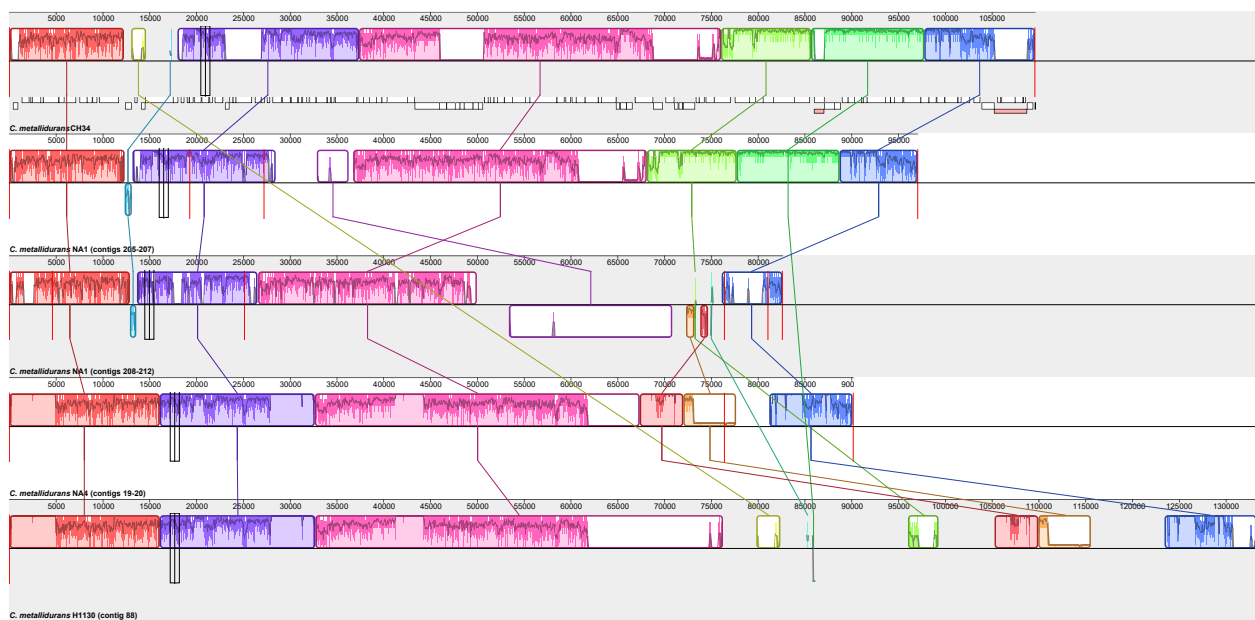

Supplement: Supplementary file 1 [file genes-09-00507-s001.zip › S2_Figure.pdf]
